# How Strong is Local Politics’ Grip on Local Economic Development? The Case of Swiss Small and Medium-Sized Towns

**DOI:** 10.1177/10780874211056519

**Published:** 2021-11-30

**Authors:** Stefan Wittwer

**Affiliations:** 1 Spatial Development and Urban Policy (SPUR), 27219 ETH Zurich, Zurich, Switzerland

**Keywords:** small and medium-sized towns, local politics, local economic development, regionalism, multilevel governance

## Abstract

Economic development directly manifests itself in the form of employment at the local level. This paper examines the ability of local politics to shape this development in a competitive federalist environment by examining how local party–political developments affect local economic development in Swiss small and medium-sized towns (SMSTs). Local economic development in the form of employment is a central local policy domain in federal and polycentric Switzerland. This paper argues that party–political influence is conditional on the characteristics of four distinguished economic sectors that differ in their dependence on the regional context. By analyzing the panel data of all Swiss SMSTs, the paper finds that local party–political developments only systematically precede growth in the residential economy, while regional processes determine the economic sectors in ambiguous ways. The grip of local politics on the development of export-oriented economies therefore is not guided by party–political development and more influential at regional levels.

## Introduction

While regional development and multilevel governance often dominate the discourse on economic development (Ansell 2002; Tödtling and Trippl 2005), this economic development directly manifests itself at the local level in the form of jobs and local economic development, thereby remaining crucial for local politics. This paper analyzes the ability of local politics to shape this development in a competitive federalist and polycentric environment by examining if party–political developments in the local executive can affect local economic development.

The focus of this paper lies on a single functional category of municipalities: small- and medium-sized towns (SMSTs). SMSTs have traditionally been neglected by urban and rural research because they fall between the fields of regional/peripheral development literature, classical urban studies and research on agglomerations. However, as Bell and Jayne (2009, 691) argue in their plea for a greater focus on smaller cities, that SMSTs act as “important nodes in the networks between places of different scales, and they are seen to mediate between the rural and the urban, as well as between the local and the global.” Additionally, as Dijkstra, Garcilazo, and McCann (2013) show in a comparative study of EU15 countries, SMSTs are catching up with large cities in terms of population growth and economic performance. A better understanding of SMSTs is therefore not only of theoretical relevance, it also follows recent empirical trends.

The vast literature on regional and multilevel governance discusses the importance of coordination and steering in upper level institutions (Pierre and Peters 2000; Savitch and Kantor 2002; Kantor 2006). With the increasing mobility of goods, people, and identities, local developments have an impact on and are impacted by developments beyond their territorial boundaries (Brenner 1999). The role of autonomous local municipalities in shaping *local* development somehow got out of sight. However, local autonomy remains a crucial feature of federalist and decentralized states where participatory processes (such as local elections) remain fragmented among local institutions (Kübler 2015) and local politics often respond to very local questions. Switzerland provides an ideal case for studying the relevance of local politics given its high fragmentation and local autonomy (Sellers and Lidström 2007). This paper views local politics as the political power constituted in the party composition of the local government in Swiss SMSTs. Different party compositions can lead the local government to take different concrete strategic and operational actions in local economic development. While this paper does not measure such strategies and actions, it is a first attempt to examine if a change in party compositions of local governments precedes changes in local economic development.

Local economic development is an especially important policy field in Switzerland’s fragmented, polycentric urban structure that rests on autonomous municipalities. Municipalities have their own budget, bear many costs, spend money at their discretion, and guarantee the provision of local public goods for their inhabitants. This financial autonomy rests on the acquisition of raising sufficient income through taxation (Linder and Mueller 2017). Consequently, SMSTs compete with many other municipalities for jobs and capital. As a result, understanding the grip of local politics is also crucial for the field of economic development policy, which often takes a regional perspective. SMSTs, as units of analysis of regional economic and political importance, thereby offer an optimal case for studying the role of development in local party politics. If we find no evidence for the assumption that local party–political development precedes change in local economic development in SMSTs, it would be likely that there is also no impact in smaller municipalities of less regional importance.

One crucial aspect of local economic development is local employment. Job growth leads to greater corporate tax income and can help to boost the importance of SMSTs in a larger region. However, SMSTs can have different types of economic specializations and develop differently. The absolute number of people employed could potentially conceal different mechanisms that underlie employment development. This paper therefore acknowledges the role of the characteristics of specific economic sectors by distinguishing between four sectors whose demand differs on their dependence on factors beyond their municipal border and, consequently, on the regional context.

This paper argues that the impact of local party–political developments is conditional on the characteristics of the employment specialization. The more dependent the economic sector is on external demand, the more important spatial context characteristics are, such as proximity to a metropolitan centre or to prospering clusters. The more the economic sector relies on the demand of its inhabitants, the more important local characteristics are, such as political conditions, population size, or local economic conditions. The ability of party–political development to explain different local economic development therefore is supposed to depend on the degree of interdependence of economic sector development.

We analyze panel data with a hybrid panel model to examine the temporal effects of local party–political development and other local and regional variables. The paper shows that it is crucial to differentiate between economic sectors to obtain a more nuanced view of local and regional factors that affect economic growth, as the results between the sectors vary considerably. A change in the party–political composition of local governments (i.e., an increase of left parties) only precedes growth of the residential economy. The effects of regional developments (measured as the development of employment in neighbouring SMSTs, cantonal growth, and the proximity to a metropolitan centre) are more ambiguous. While the development of employment in neighbouring SMSTs and the proximity to a metropolitan center have the greatest impact on the development of the heavily export-oriented knowledge-intensive business sectors (KIBS), the development of KIBS is not systematically related to cantonal growth, and regional developments also affect the other economic sectors. The results indicate that different party compositions in the local executive can lead to different local economic policy strategies that aim to support the residential economy. The paper does not measure different local economic development strategies and how they are related to political parties. However, we discuss how party politics in the residential economy can manifest itself in Swiss SMSTs and how politics’ local grip on the development of export-oriented economies (a) is not guided by party–political development and (b) is more influential by activity at the regional and urban level.

In terms of the question of local “post-political consensual governing” (Peterson 1981; Swyngedouw 2009), the findings indicate that an economic sector’s degree of export orientation influences how local factors are able to shape economic development. Additionally, the findings contribute to the literature on multilevel governance by including spatial factors to address questions of local autonomy in a federalist and decentralized setting (Sager 2005; Koch 2013; Kübler and Rochat 2018; Wittwer 2020).

The structure of the paper is as follows. The next section discusses the role of local politics in local economic development and derives the hypotheses. Then, there is a description of the data, the data-gathering process, and the hybrid within-between random effects model. Afterwards follows the presentation of the results of the analyses on four different economic sectors and a discussion on the role of local party–political strategies on economic development. In the conclusion, we sum up the paper and discuss its implications, contributions, and further steps of research.

## Local Politics and Local Economic Development

### The Phenomenon to be Explained

Research on decentralized countries shows that local parties can matter for local revenue policies such as local income and property taxes and local public spending (Einstein and Kogan 2015; Kübler and Rochat 2018; Egerod and Larsen 2021). This is also true in Switzerland where tax rates and the economic freedom of local enterprises are two of elected local officials’ main self-reported issues (Geser et al. 2011; Geser and Meuli 2011). Economic development policy, however, is not a classical local policy field because much is negotiated on higher regional levels (Ansell 2002). Nonetheless, empirical studies on SMSTs and smaller municipalities in Switzerland show that they not only use their discretion in designing local tax policy (Brülhart, Jametti, and Schmidheiny 2012) and land-use policy (Devecchi 2016; Berli 2018; Kaufmann and Meili 2019) but also that these policies have the potential to shape local economic development (see also Kaufmann and Arnold 2018). Additionally, regional governance and vertical exchange with cantonal entities enable local politicians to advocate for their municipality. As regional development organizations generally operate through cooperation agreements that are based on contracts that do not include directly elected representatives and formal democratic procedures at the regional level (Wittwer 2020), the public can only express their demands through local elections and votes. While the paper does not contest the relevance of regional governance and inter-municipal cooperation for local and regional development, the extent of the role of local autonomy remains a crucial question, particularly in times of decreasing public interest in local politics (see Kübler 2015, 14).

Local elections often are considered as being elections of personalities and experience and not particularly of parties and ideologies (Jennings and Niemi 1966; Marien, Dassonneville, and Hooghe 2015). For members of local executives in Swiss SMSTs, independent of the size of the municipality, reputation is the most important (self-reported) requirement for getting elected. Party affiliation has been important for only 31.75% of elected officials while factors such as orderly family relationships and high professional qualifications were similarly important requirements for getting elected (Geser et al. 2011, 90; Geser and Meuli 2011).

While a crucial goal for the elected officials surveyed irrespective of their party (range 80–90%) is attracting wealthy residents to increase the tax base and demand in the local economy, the strategy of lowering tax rates to attract wealthy tax payers is substantially more disputed at the local level (range 34–88%) (Geser et al. 2011, 65). Lowering tax rates is therefore not the only instrument for attracting wealthy residents. Geser and Meuli (2011, 10) also show that fostering economic growth is mainly a crucial goal for right and middle parties (for 52–58%) but also for 41% of local officials of left parties.

### The Role of Local Party-Politics in Local Economic Development

These numbers indicate that parties are not the only crucial factor in the local elections of SMSTs: they are one of many factors. Once elected, however, party affiliation becomes important: local officials from left parties state different aims and focal points for economic development strategies than those of more economic liberal middle and right parties. The focus of this paper is to capture the role of local party–political development in local economic development in an explorative way to understand if party–political developments precede a change in local economic development. However, literature on local politics also discusses how different parties have different local development policy strategies.

From an ideological point of view, left parties have a stronger focus on redistribution than on economic growth (Geser 2009; Hajnal and Trounstine 2010; Einstein and Glick 2018). The role of the city is to use its capacities to leverage endogenous assets such as land and labour to build a more socially just and self-sustaining path to local economic development. The focus lies on the role of essential workers and local jobs that focus on local assets instead of inter-municipal competition and positioning strategies to attract external investment or creative human capital (Imbroscio 2013, 1995; Joy and Vogel 2021, 12; Thompson et al. 2020).

The empirical literature on the impact of political ideology on local policy-making has a strong focus on U.S. municipalities and on public spending and taxes. Gerber and Hopkins (2011) find that party affiliation of mayors in U.S. cities does not affect public spending in policies that can be linked to urban development. Einstein and Glick (2018) show in a survey with mayors that Democratic mayors are more likely to support and implement redistributive policies, while Sances (2021) shows that more Democratic votes increase public spending and revenues in the United States. Hajnal and Trounstine (2010) show that politics has an impact on local spending, but, in line with Peterson (1981), also do economic constraints, institutions and economic interest groups.

The two-party system of the United States is difficult to compare to European countries with more parties and a more pronounced tradition of left parties. Analyzing Bavarian municipalities, Freier and Odendahl (2015) show that the green party raises property taxes and the pro-market liberal party lowers them, while the center–left social–democratic party is found to lower taxes on firms.

Next to tax policy that can increase local revenues and spending or attract firms with low business tax rates, another way to shape economic development is through land-use planning, for example, more active land policy where municipalities can formulate conditions for planning and land use (Kaufmann and Meili 2019). Solé-Ollé and Viladecans-Marsal (2013) show that in Spanish municipalities, left-wing governments on average allow less land to be developed than right-wing governments. As Devecchi (2016, 361–362) shows for Swiss municipalities, liberal members of local executives do not favour measures that are too interventionist. However, Devecchi (2016) also argues that economic conditions and the degree of professionalization are more important for explaining land-use policy strategies than party affiliation.

The finding by Geser et al. (2011) that attracting wealthy residents to increase the tax base and demand in the local economy is shared among elected officials irrespective of their party, but the means to attract them with low tax rates is disputed (Geser et al. 2011, 65) also points to the direction of different partisan development strategies.

### A Differentiated Approach on Local Autonomy in Local Economic Development

Although party membership is not central to local elections, it shapes preferences for the design of local policies and therefore can result in different policy outcomes. Local politicians who favour economic growth are more likely to advocate for policies that foster growth and to advocate for the economic interests of their municipalities in upper-level institutions or cooperation arrangements (Feiock 2007; Feiock, Steinacker, and Park 2009; Bel and Sebő 2021). However, the more a sector that is important to the local level depends on resources and demands that the SMST cannot provide autonomously, the more restricted party–political aims will be.

This paper examines whether local party politics can explain local economic development or whether upper level, regional or national developments determine it. While the former process would highlight the ongoing importance of local politics and fragmented authority, the latter would indicate processes of regional determinism or “managerial consensual governing” (Swyngedouw 2009, 605), where party politics has a low impact. Regional determinism means that local political parties are not able to shape local economic policy because regional and national developments are stronger and decisive (Gerber and Hopkins 2011; Kaufmann and Wittwer 2019). Different cleavages based on a different reliance on local assets of the economic sectors (Hiscox 2001) then are supposed to manifest themselves only on regional or even higher levels. The post-political condition of “managerial consensual governing” indicates that there is a local political party consensus on an entrepreneurial stance to local economic development, which means that all parties want to engage in inter-municipal competition in the same way to compete for resources (Peterson 1981; Harvey 1989, 4–5; Jensen and Malesky 2018; Filion, Reese, and Sands 2019). As Peterson (1981) argues, local politics matter only for allocative policies because these policies have no impact on the economic position of the city. In development and redistributive policies, there should be a consensus between parties.

These processes of regional determinism or “managerial consensual governing” may emerge even stronger in the case of SMSTs that lack the “centrality of cities for public contestation” (Beveridge and Koch 2016, 41). While some SMSTs are regional centres of economic relevance, they have fewer amenities (cultural, sports, universities and so on) to offer to their inhabitants than larger cities, and they have fewer options for designing local policy in various ways. Additionally, spatial barriers between SMSTs are lower than between cities (because there are more SMSTs than cities), and they directly contend with municipalities that are more similar. As a result, it is possible to assume that competition for “development capital” (Harvey 1989, 11) is even more intense. A recent study by Kübler and Rochat (2018) on intergovernmental cooperation and revenue sharing in local social policy supports this assumption and finds that local party politics only impacts social expenditures in metropolitan core cities, not in metropolitan municipalities.

However, the focus here does not lie on the impact of local party politics on expenditures, but on the ways to *acquire* money through economic development: Can local party politics explain local economic development? We argue that local politics can still affect economic development but that this influence depends on the characteristics of different economic sectors (Alt et al. 1999; Hiscox 2001). Additionally, the location of an SMST supposedly impacts local politics: It is likely that local politics in SMSTs that are close to metropolitan centres in agglomerations follow external developments (Meijers and Burger 2017) while more remote SMSTs are more autonomous when designing their economic policy.

The basic argument is that local party–political developments can affect economic development but that economic development requires a more differentiated approach. Although literature on polycentric urban regions assumes strong interdependency (Meijers and Burger 2017; Wittwer 2020), a differentiated view on economic sectors can address different extents of interdependence. Consequently, the impact of local party–political developments relies on the characteristic of the employment specialization (Box 1 summarizes the hypotheses).

**Figure 1.**
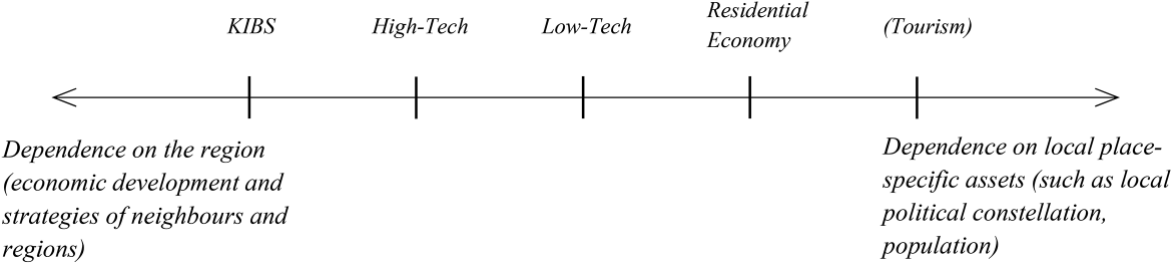
Relation of economic sectors to spatial context (own illustration). KIBS: Knowledge-intensive business services.

As Figure 1 illustrates, economic sectors that are more to the right of the line are more dependent on local place-specific assets while sectors more to the left are more dependent on developments in the broader region. The figure includes the tourism sector as an example of a sector that is heavily dependent on place-specific assets (such as the landscape); however, it does not figure in the paper.

The residential economy and low-tech (LT) industries are more immobile and place-bound factors are important to them, and they are not so dependent on regional developments. The residential economic sector offers goods and services for the local demand. This sector exists in every SMST to some degree (i.e., hairdressers, wholesales, bakeries). The LT industry relies on large, immobile manufacturing infrastructure and often exploits natural resources that are place-bound and are therefore also less mobile (Alt et al. 1999). The local autonomy for shaping these sectors is larger and local party–political development should have a visible effect on them. Additionally, firms that rely on local immobile assets are more likely to lobby for local political support (Alt et al. 1999). As the residential economy is not primarily export-oriented, the effect of local place-specific assets is likely more substantial than in the LT industry. High-tech (HT) industries and KIBS are classical export-oriented economies that rely on specialized knowledge that can be found in a broader region. Similar to LT industries, HT industries can rely on immobile manufacturing infrastructure, but they are less dependent on place-bound resources—they are more mobile than LT industries but less mobile than KIBS. KIBS primarily rely on knowledge and talent and do not require immobile infrastructure. As a result, local development is less important than what takes place in the SMST’s region. However, given that HT industries rely more on infrastructure and are less flexible in changing location, they rely more on local assets than KIBS.

**Box 1**
***H1:*** *For the development of the residential economy, local characteristics are more important than regional characteristics.*
***H2:*** *For the development of the LT sector, local characteristics are more important than regional characteristics, although they also play a role.*
***H3:*** *For the development of the HT sector, regional characteristics are more important than local characteristics, although they also play a role.*
***H4:*** *For the development of KIBS, regional characteristics are more important than local characteristics.*

## Research Design

### Data

The paper focuses on SMSTs in Switzerland. The Swiss Federal Statistical Office (FSO) defines SMSTs based on European Definitions (Dijkstra and Poelman 2012). It defines an SMST as a minimum of 14,000 inhabitants, workers or overnight stays and as having a dense core with at least 2500 inhabitants, workers or overnight stays per square km (see Goebel and Kohler 2014, 15).

Data on the dependent variable, the *development of employment in different economic sectors* in SMSTs in Switzerland, includes people working in a specific sector as full-time employment equivalence in each SMST for the years 2005, 2008, 2011, 2012, and 2013 (in addition to 1995 and 2001 for time-lagged variables).^1^ The sample consists of five periods. The four sectors are (a) KIBS that are reliant on high professional knowledge, such as scientific research, computer programming, consulting, legal activities), (b) HT industries such as the pharmaceutical industry, electronic devices, (c) LT industries (manufacturing of paper, textile, cement), and (d) residential economies ((RES) such as reparations, wholesalers, hairdressers, construction) (see Meili and Mayer 2017).

Based on the classification by Eurostat (2008), Meili and Mayer (2017) assign each firm in an SMST to one type of economic structure and measure the amount of full-time equivalency in each sector of each SMST.^2^ A one point increase in full-time equivalency thus equals one more full-time employment position in an SMST in the respective economic sector.

Table 1 displays some descriptive information on Swiss SMSTs (see the Appendix for a full operationalization table). SMSTs are on average more conservative than cities (around 70% non-left parties (SMSTs) vs. 42% non-left parties (cities) in the local executive) and consist of around 35% of the Swiss population. Meanwhile, the ten larger cities consist of around 17% of the Swiss population (FSO, see Appendix for a full operationalization table).

**Table 1.** Descriptive Data on Swiss SMSTs (Averaged Over Years and Units).

	Mean	*SD*	min	max
Full employment	7,671	4,860	1,356	26,138
Residential	5,602	3,820	1,306	19,313
KIBS	528	679	9	5’795
Low-tech	708	541	14	3717
High-tech	833	1063	0	7’287
Share of non-left parties in executive	72%	17%	27%	100%
Population	14,894	6,879	5,268	41,157

*Note.* KIBS = knowledge-intensive business sectors; SMST = small and medium-sized towns.

A self-extended dataset from the FSO identifies the composition of the local government of SMSTs, including the left/right dimension of parties in local governments. Local governments as the local executive branch have three to 30^3^ seats (6.7 on average for SMSTs). Even though there is an elected president, the government is based on the principle of collegiality and consociationalism (Geser et al. 2011). While there is comparative research on local politics in Switzerland that relies on the readily available data of local vote shares in national parliamentary elections (Berli 2018; Kübler and Rochat 2018; Kaufmann and Wittwer 2019) or on a sample of survey respondents (Geser et al. 2011), this study is, to the best of the authors’ knowledge, the first that incorporates data on the local executive level for all SMSTs over time. Local vote shares in national parliamentary elections are good proxies for measuring local political preferences because they capture the political preferences of the local population (those allowed to vote). However, it does not provide much information on local political action and power structures. The election of the local executive does not exactly represent the local political preferences in elections.^4^ When focusing on local party–political power structures to examine the impact of local politics, it is more accurate to measure the composition of the local executive.

Since the number of towns in the previous FSO definition steadily increased, data on earlier years do not include all SMSTs of interest. To ensure the analysis of the full sample of all Swiss SMSTs, we extended the data set by the FSO and collected data on the composition of the local governments with the help of a comprehensive media analysis. For each SMST not included in the FSO’s sample, we analyzed official municipal online documentation, and, in the common case of no online documentation, newspaper articles following local elections^5^ to find information on local election results. Similarly, we collected information on independent local officials to situate them along a left/non-left dichotomy. We code these officials as zero if they position themselves to the left or if they were supported by left parties (Social Democratic Party of Switzerland (SPS); Green Party of Switzerland (GPS); Alternative Left Party of Switzerland (AL); Christian Social Party of Switzerland (CSP) and other local left parties^6^) and as 1 otherwise.

To take into account the spatial environment of SMSTs, we rely on two variables that take into account development in neighbouring SMSTs. First to measure a spill-over effect, we use a self-coded dataset that incorporates the spatial context of SMSTs. A binary matrix (C) specifies ‘connectivities’ between all SMSTs and assigns a 1 to the connectivity (*c_ij_)* of SMST *i* and *j* if a connection exists and a 0 if there is no connection (Ward and Gleditsch 2008, 14; Neumayer and Pluemper 2016). If an SMST has a connection with another SMST or a city within a driving distance of less than 20 min^7^, we measure it as spatially proximate and we consider the two SMSTs (or a SMST and a city) to be neighbours. The binary matrix (C) then shows which SMSTs are connected and the amount of other SMSTs and cities each SMST is connected to. It also calculates the spatial lag of development in neighbouring SMSTs.

We measure the spatial lag of the economic development of neighbouring towns by examining the amount of full-time equivalent jobs of its neighbours. By measuring the amount of full-time equivalent jobs of an SMST’s neighbours at *t −* 1 and adding them up, we can assess the impact of the economic development of SMSTs close to the SMST under observation. We can therefore measure whether the increase or decrease of full-time equivalent jobs of neighbouring SMSTs in previous years (at *t − 1*) impacts the development of a specific economic sector of the SMST under observation. This spill-over variable enables us to quantify the impact of developments in neighbouring SMSTs.

For the second variable that takes into account regional development in a broader sense, we measure economic growth at the cantonal level in the form of the development of the canton’s gross domestic product.^8^ Cantons are important actors in economic development policy in Switzerland (Kaufmann and Meili 2019; Wittwer, Sager and Huegli 2021). Including cantonal growth allows examining whether economic performance at higher institutional levels shape employment development at lower levels.^9^

To measure the role of large cities, we measure how close a SMST is to large metropolitan centres. We assess the distance in train minutes for each SMST to its nearest metropolitan centre (Zurich, Geneva, Lausanne, Basel, Bern, Milan (Italy)). Data on population are taken from the Swiss FSO (see operationalization table in the Appendix).

### Methods

This paper uses a hybrid panel data approach that combines fixed and random effects ‘that yields the advantages of fixed effects while identifying the parameters of time-invariant regressors’ (Halaby 2004, 530). This means that we mix the properties of fixed effects for the time-varying explanatory variables with random effects estimators for stable explanatory variables (see also Mundlak 1978; Bell and Jones 2015). In this so-called ‘hybrid model’ (Halaby 2004) or ‘within-between Random Effects’ (Bell and Jones 2015), we estimate within effects in a random effects model by decomposing time-varying variables into a between $\left({\bar{x}}_{i}\right)$ and a within $\left(x_{it}-{\bar{x}}_{i}\right)$ component. The within component, which only measures the change over time (e.g., change in the political composition of the executive), is a pure fixed effects estimator that avoids unit heterogeneity bias. The time-invariant between component and the other time-invariant variables (e.g., the average share of non-left parties in the executive over the years or cantonal membership) are random effect estimators interested in the variation between the observations and can therefore can still be subject to heterogeneity bias if important variables are missing (Halaby 2004, 531).

We measure the independent variables at time *t − 1* to track how past developments impact the current outcome of interest (e.g., how party-political development at time *t − 1* affect jobs in a specific sector at time *t*). The lag of the dependent variable is measured at *t − 2*.^10^ We use panel-robust standard errors for SMSTs to control for serial correlation and heteroscedasticity (Halaby 2004, 524). To control for outliers, the dependent variable as well as the lag of the dependent variable, the population variables and the spatial lags are logged (see Operationalization table in the Appendix).

One important point for this paper is to make sure that party–political development is not endogenous to local economic development. As we argued in the theory section, local elections often are considered as being elections of personalities and experience and not particularly of parties and ideologies (Jennings and Niemi 1966; Marien, Dassonneville, and Hooghe 2015). Literature on the political preferences of voters indicates that their preferences are very stable and governed by long-term values that do not often change in volatile economic circumstances (see e.g., Sears and Funk 1999; O’Grady 2019). Additionally, research from the United Kingdom and Sweden shows that local economic conditions and tax rates do not impact local voting decisions (James and John 2007; Elinder 2010). A change in the political composition of the executive is therefore not often due to party–political competition regarding local employment development, but it is instead influenced by external developments such as withdrawals and new popular candidates from different parties in elections. Table A5 in the online Appendix also tests this assumption empirically by regressing party–political development on prior economic development in the different economic sectors and finds no signs of endogeneity. In other words, the regressions show that local party–political change does not depend on prior economic development in Swiss SMSTs.

The next section presents the results of the hybrid panel models for the four economic sectors.

## Results

Table 2 displays the results of the hybrid panel models for the four economic sectors using two different measures for regional development.^11^ The ‘within’ variables account for the fixed-effects estimates, that is, the development over time for each SMST. The ‘between’ variables account for the comparison of the values between the SMSTs. The results discuss each economic sector separately before comparing them in the discussion section.

**Table 2.** Results of Within–Between Random Effects Models.

	(1.1)	(1.2)	(2.1)	(2.2)	(3.1)	(3.2)	(4.1)	(4.2)
	Residential	Residential	Low-tech	Low-tech	High-tech	High-tech	KIBS	KIBS
Prior development	0.135***	0.144***	0.121**	0.118**	0.0495	0.0502	0.0928	0.0990
in the sector (*t* − 2)	(0.0372)	(0.0380)	(0.0575)	(0.0567)	(0.0870)	(0.0877)	(0.0598)	(0.0607)
Party composition	−0.101	−0.104	−0.157	−0.158	−0.941	−0.942	−0.568	−0.581
(*between*)	(0.342)	(0.342)	(0.630)	(0.629)	(1.146)	(1.148)	(0.639)	(0.642)
Party composition	−0.0798**	−0.0837**	−0.291	−0.280	0.184	0.166	−0.0742	−0.0936
(*within*)	(0.0364)	(0.0366)	(0.179)	(0.180)	(0.272)	(0.270)	(0.191)	(0.192)
Economic development of	0.114**		−0.0224		0.0613		0.400**	
neighbours (*within*)	(0.0422)		(0.115)		(0.242)		(0.193)	
Cantonal economic		0.0762		0.236*		−0.378*		0.175
development (*within*)		(0.0521)		(0.137)		(0.219)		(0.219)
Distance to metropolitan	−0.0698**	−0.0701**	0.00308	0.00315	0.00988	0.00970	−0.213***	−0.214***
Centre (*between*)	(0.0236)	(0.0236)	(0.0360)	(0.0360)	(0.0829)	(0.0828)	(0.0390)	(0.0389)
Population	0.971***	0.970***	0.697***	0.698***	0.630**	0.630**	1.078***	1.076***
(*between*)	(0.0905)	(0.0905)	(0.150)	(0.150)	(0.308)	(0.308)	(0.173)	(0.173)
Population	0.351***	0.366***	0.234	0.248	0.854*	0.834*	0.535**	0.600**
(*within*)	(0.0874)	(0.0963)	(0.221)	(0.228)	(0.479)	(0.455)	(0.268)	(0.289)
Cantonal^a^ and year fixed effects	Yes	Yes	Yes	Yes	Yes	Yes	Yes	Yes
Intercept	8.060***	8.087***	5.760***	6.013***	6.484***	6.329***	6.285***	6.343***
	(0.319)	(0.318)	(0.209)	(0.562)	(0.975)	(0.972)	(0.594)	(0.605)
*N (SMSTs/years)*	740 (148/5)	740 (148/5)	740 (148/5)	740 (148/5)	740 (148/5)	740 (148/5)	740 (148/5)	740 (148/5)
*R^2^ within*	0.77	0.77	0.07	0.07	0.02	0.02	0.24	0.23
*R^2^ between*	0.58	0.57	0.35	0.34	0.27	0.25	0.42	0.41

Panel-robust standard errors in parentheses.

* *P* < .1, ** *P* < .05, *** *P* < .001. Residential = Residential economy; KIBS = Knowledge-intensive business sector.

a Cantonal fixed effects are only applied for the between-effects.

### Residential Economy (Models 1.1–1.2)

The models that examine the development of the residential economy reveal the influence of local development, and particularly that of local politics. An increase in the share of nonleftist parties in the composition of the local government led to a decrease in jobs in the residential sector. A 20% decrease (i.e., a one-seat change in a five-person executive) resulted in an average 1.7% decrease in the number of full-time-equivalent jobs in the residential economy, all else remaining equal. This indicates that a turn to more leftist parties in SMST’s local governments results in a greater focus on promoting the residential economy.

If neighbouring SMSTs grew into more of an export-oriented economy, the residential economy in the respective SMST on average grew too. Additionally, previous development in the residential sector, larger and growing SMSTs and SMSTs closer to a metropolitan centre experienced more job growth in the residential economy. The results corroborate the first hypothesis, which postulates that local factors play a central role in determining local economic development. However, while cantonal growth does not have a significant effect on the residential economy, the development in neighbouring SMSTs and the distance to the metropolitan centre show that regional effects also hold true for the residential economy.

### Low-Tech Industry (Models 2.1–2.2)

The results are different for the LT sectors. The R^2^ is much lower, which indicates that the variables in the model only weakly explain SMSTs’ variance in economic development. Only a higher population on average, prior development and cantonal growth lead to an increase in development in this sector. Developments in the medium and LT industry were therefore neither guided by specific party constellations nor by the developments of neighbouring SMSTs or the metropolitan centre. Possibly, idiosyncratic factors mainly drove development. Idiosyncratic factors can include the path-dependent growth of manufacturing firms established long before the period examined by the data. As the manufacturing industry relies more on infrastructure (large buildings for processing goods, such as wood), it is expected to be less flexible than residential economies and KIBS. Consequently, the results corroborate hypothesis 2 given that only local characteristics play a role in job development in the LT industry.

### High-Tech Industry (Models 3.1–3.2)

The development of the HT sector is even more difficult to compare with an even lower *R*^2^. Prior development in the sector has had no systematic effect; only a larger population on average and over time and cantonal development seem to contribute to the explanation of job growth. However, the effect of cantonal growth is counterintuitive: In cantons that increased their economic performance, employment in the HT industry in SMSTs, on average, decreased. We find no evidence for local political factors and regional development. Once again, development in this sector possibly is mainly driven by idiosyncratic factors. Similar to the LT industry, idiosyncratic factors can be the path-dependent growth of large firms manufacturing highly specialized goods that require large buildings such as a pharmaceutical laboratory and could explain the counterintuitive relation to cantonal growth. Even though the HT sector is more dependent on mobile knowledge than the LT sector, local infrastructure remains crucial, and we therefore reject hypothesis 3.

### Knowledge-Intensive Business Services (Models 4.1–4.2)

As for the residential economy, the model fits better to describe the development for KIBS than LT/HT sectors. As postulated, we find no evidence for a systematic effect of party political factors, i.e. employment in KIBS has developed similarly in SMSTs with either a high or a low share and increasing or decreasing rates of non-left parties. Regional factors, however, seem to play a more important role: The economic development of neighbouring SMSTs has a strong positive effect on local economic development while prior development in the SMST of observation does not. If employment in the export-oriented sector in a neighbouring SMSTs rises by 10%, the number of full-time-equivalent jobs in KIBS grew by an average of 4%. Additionally, the effect of being more remote from a metropolitan centre is also strongly negative, which indicates that the further away an SMST from a metropolitan city, the lower is the employment development in KIBS (around 20% less for 10 min). Interestingly, cantonal growth does not systematically affect the development of KIBS, indicating that the regional drivers for KIBS are metropolitan centres.

Additionally, the results show that the development of KIBS also depends on local-specific developments because the size and trend of the local population have a strong positive effect. The results corroborate hypothesis 4 because regional, or, more precise, urban characteristics prove crucial for development. However, the influence of the size of the local population also indicates that local characteristics are also important.

### The Role of Different Economic Sectors

**Figure 2.**
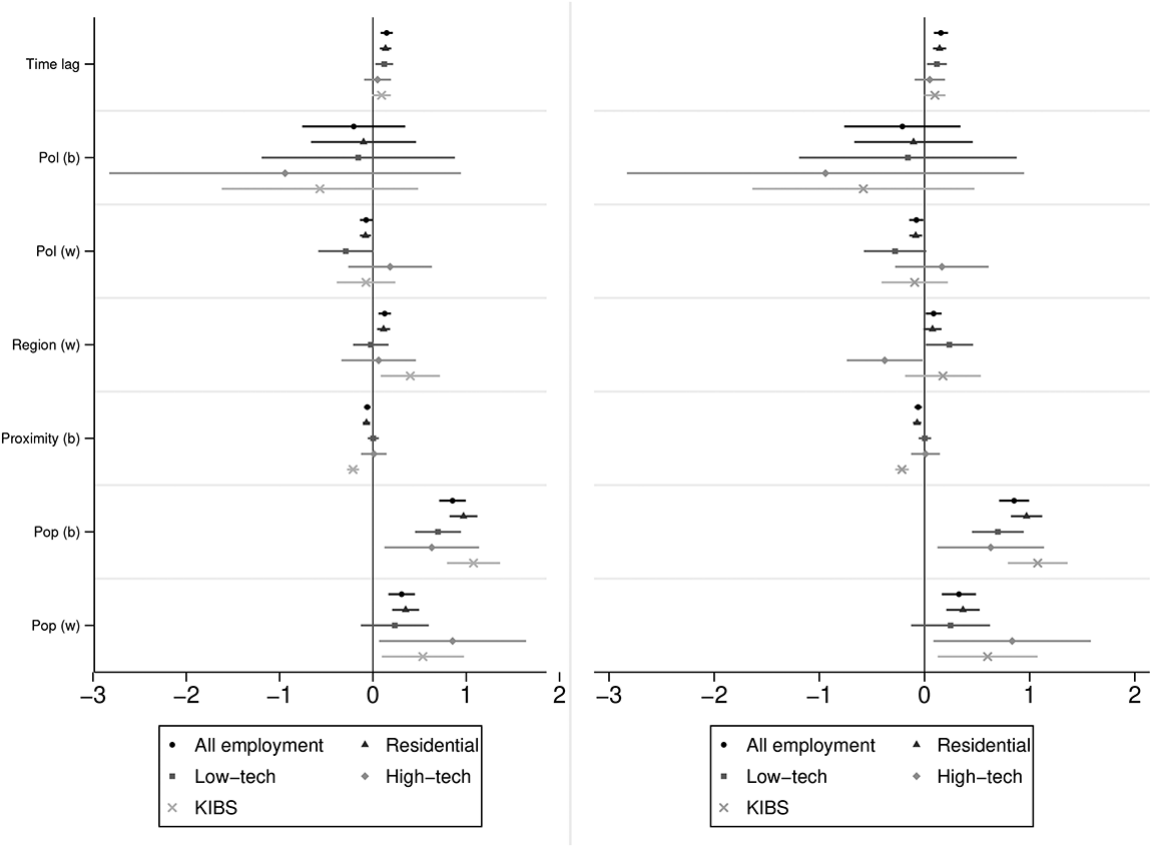
Coefficient plot for the eight models on different economic sectors. Legend: Left for variable measuring neighbouring developments as regional effect. Right for variable measuring cantonal development as regional effect. (w) for within effects, (b) for between effects; "Pol" displays the coefficients for party composition.

The results support the assumptions that a differentiated view on economic development contributes to a better understanding of the potential for local politics to shape it. This section contrasts the results of different economic sectors on development before discussing local politics’ grip on economic development. Figure 2 compares the results using two coefficient plots that display the estimates of the variables of the models of Table 2 and their confidence intervals at the 90% level.^12^ For each variable, the figure compares the coefficients of all four economic sectors and the coefficient of a model that added all economic sectors (i.e., total employment, see Table A3 in the online Appendix).

The comparison shows that it is fruitful to differentiate between economic sectors to obtain a more nuanced view on local and regional factors that enable or impede economic growth. The results between the sectors vary considerably and the coefficients of the model with the full employment conceal these differentiated effects.

In line with the assumptions, local government composition on the one hand only plays a role in the development of the residential economy. The coefficients manifesting the average effect of party–political development are similar or even larger in the LT sector and KIBS, but the standard errors are much higher and the confidence interval includes zero even on the 90% confidence interval (see Figure 2), especially in the HT sector and KIBS. Interestingly, in the LT sector that also is supposed to rely more on local assets, the comparatively high negative average effect indicates that SMSTs with an increase in the share of nonleftist parties in the composition of the local government led to a decrease in jobs in the LT sector even more substantially than in the residential sector. However, the high standard error shows that there are also SMSTs with a converse effect.

On the other hand, regional developments (measured as the development of employment in neighboring SMSTs and the proximity to a metropolitan centre) have the greatest impact on the development of KIBS. SMSTs surrounded by other SMSTs that have experienced job growth and are closer to metropolitan centres experienced more job growth in KIBS. However, cantonal growth as another measure for regional development does not affect job growth in KIBS. This indicates that regional determinants on KIBS mainly are the embeddedness in metropolitan areas and consequently, proximity to larger cities. Additionally, cantons can promote development by putting emphasis on certain centre regions (Kaufmann and Meili 2019). These findings support the assumption that KIBS benefit most from network knowledge, information exchange, and pecuniary and technological spill-overs, while LT- and HT industries rely more on local idiosyncratic factors than on regional spill-overs. Additionally, prior development has no impact on the more export-oriented sectors of KIBS and HT. However, the case for the HT sector is less clear and manifold causes could drive development in the sector. Future research should examine this using a more inductive, case-based approach.

These regional effects also hold true for the residential economy, although at a lower substantive level. For residential economies, prosperous neighborhoods with a growing export-oriented economy and to larger cities increase the demand for residential goods, such as restaurants and department stores as people might otherwise commute to neighboring SMSTs. The residential economy is therefore not only driven by local place-specific assets but also by its regional environment.

**Table:** 

**Box 2**	**Findings**
***H1****: For the development of the residential economy, local characteristics are more important than regional characteristics.*	***Yes, but also regional characteristics***
***H2****: For the development of the LT sector, local characteristics are more important than regional characteristics, although they also play a role*.	***Yes, but also regional characteristics***
***H3****: For the development of the HT sector, regional characteristics are more important than local characteristics, although they also play a role*.	***Yes, but also local characteristics***
***H4****: For the development of KIBS, regional characteristics are more important than local characteristics.*	***Yes, but especially proximity to centers and also local population***

## Discussion: Local Politics and Local Economic Development

These findings shed light on the question of whether the different party–political compositions of local executives can shape local economic development. While surveys with members of local governments show that party–political differences exist when assessing economic growth as a crucial policy goal, this paper finds that a different composition in the local executive over time only precedes growth in the residential economy and that the effect is rather low compared with other factors. Local governments composed of a higher share of left parties are more successful in or focused on promoting the residential economy.

For further analyses, we also included three dichotomous variables to the data that take into account different institutional properties that can influence the autonomy of SMSTs (Lubell, Feiock, and De La Cruz 2009). One that measures whether the SMST has an elected parliament as legislative branch or a municipal assembly (from Ladner 2016), one that measures whether the SMST belongs to the French-speaking part of Switzerland, where local autonomy is traditionally lower (see e.g., Linder, Zürcher, and Bolliger 2008) and one that measures if an SMST is more than 20 min remote from a metropolitan centre and hence has more autonomy (see e.g., Kübler and Rochat 2018). I interacted the dummies with the variables on the local party–political composition in the executive to find out whether the effects of local party–political development are stronger in SMSTs with parliaments or in SMSTs in the non-French-speaking SMSTs with traditionally higher local autonomy or more remote SMSTs. However, the coefficients for all variables do not vary, as the figure in online Appendix 7 shows, indicating that the language region, the presence of a local parliament, and being more remote do not have an impact on the role of local party politics in economic development.

How does this finding relate to the literature on the impact of parties on local politics discussed in the theory section? Alt et al. (1999) show that firms with more immobile assets relied more on local political support. Hence, first, it can indicate that left parties successfully promote strategies that aim to guarantee a residential economy friendly environment, for example, by traffic calming, or by actively promoting the SMST in the region to benefit from spill-overs. Residential economies generally depend heavily on local demand, which local land-use policy can possibly better shape, while the other three sectors are export-oriented and other kinds of incentives attract them, such as low corporate income taxes and agglomeration economies (see e.g., Brülhart, Jametti, and Schmidheiny 2012). As Kaufmann and Wittwer (2019) and Kaufmann and Meili (2019) show, land-use policy is an area where the local level still has the potential to shape economic development. The findings on the residential economy indicate that the party–political composition of the local executive can lead to different land-use planning strategies or generally more progressive policies promoting the residential economy by “lessen[ing] the extent that resources and capital flow out of the local economy” (Imbroscio 1995; Filion, Reese, and Sands 2019, 17), for example, with buy-local campaigns (called “self-reliance strategies”; Imbroscio 1995). Hence, on the one hand, left parties may focus on increasing local demand by supporting self-reliance strategies or enlarging the population and gathering places instead of building large industrial zones. Berli (2018), for example, shows that in the metropolitan area of Zurich, decisions on growth control depend on the vote share of environmentalist parties (in national parliamentary elections) and Solé-Ollé and Viladecans-Marsal (2013) show that left-wing governments on average allow less land to be developed. On the other hand, our results indicate that promoting industrial zones that focus on export-oriented industries is not guided by different party–political compositions.

For the other three economic sectors, different party–political compositions do not precede specific local economic development. There are two possible explanations for these findings:

First, SMSTs do not have much leeway when implementing local strategies that aim to promote LT/HT sectors or KIBS. This could be because they are mainly driven by local path-dependent development in the case of the LT/HT industry or by developments in larger metropolitan centres in the case of KIBS (see also Gerber and Hopkins 2011). However, it does not indicate that politicians from different parties do not put emphasis on different economic development strategies, for example, that politicians from non-left parties support more tax cuts (Hiscox 2001; Geser et al. 2011; Freier and Odendahl 2015). As Jensen and Malesky (2018, 5) argue and demonstrate for cities in the United States, Canada, and the United Kingdom, politicians follow strategies to attract global firms using local incentives also if these strategies prove ineffective. As they argue in Jensen, Malesky, and Walsh (2015), “given that voters believe incentives to be an effective policy, politicians can pander to voters by using these policies, even if politicians truly believe that they are ineffective or too costly” (337).

The second explanation could be that SMSTs are able to implement local strategies; however, the decision to promote these sectors does not depend on the party–political composition of the government because of a cross-party consensus driven by economic competition (Peterson 1981; Harvey 1989). For KIBS, the substantial impact of the spatial environment is likely to be manifested by the SMST’s proximity to the metropolitan center. This indicates that urban developments are also crucial drivers of local economic development. The results show that in SMSTs with a similar regional context, different political compositions do not lead to different outcomes even though governments can decide on how proactively they want to try to benefit from regional spill-overs, for example, by engaging in regional or agglomeration institutions (see e.g., Feiock, Steinacker, and Park 2009; Bel and Sebő 2021) or by pursuing a tax strategy that is more competitive than their neighbours’ (see e.g. Brülhart, Jametti, and Schmidheiny 2012; Jensen, Malesky, and Walsh 2015). Hence, it is possible to argue that a cross-party consensus exists, and therefore also a state of post-political “managerial consensual governing” (Swyngedouw 2009, 605).

To sum up, this paper sheds light on the role of local party politics and spatial factors and how their influence depends on the export orientation and factor immobility of the economic sector. For further research on the role of local party politics in local economic development, it would be particularly interesting to examine if and how left parties differ in their strategies for promoting the residential economy and if parties formulate specific strategies for attracting more mobile KIBS, even though the prospects are questionable.

## Conclusion

This paper examines the relation of local party–political development and economic development in Swiss small and medium-sized towns (SMSTs). Based on recent literature on the role of locational policies for local development and surveys with elected local government officials, we argue that even though parties do not play a crucial role in the election of local government officials, local politicians still emphasize different local economic development measures based on their party background. Additionally, even though local economic development is a policy field where higher level entities such as cantons and regions play an important role, local politicians in SMSTs can still shape locational policies, and they can decide on how intensely they promote their SMST in these higher level entities. This is especially true given that regional engagement mostly only occurs through contractual or even informal agreements that guarantee the discretion of SMST engagement. We argue that the characteristics of four different economic sectors (residential economy, LT industry, HT industry and knowledge-intensive industry) influence how local politics affect economic development. The more specialized the sector is and the less it is based on locally specific assets, the more regional development and regional spill-overs or agglomeration effects influence it. Conversely, the more important place-specific assets are, such as local demand, the more impact local party–political developments can have.

This paper analyzes a full sample of SMSTs in Switzerland, that is, a specific category of municipalities that have sufficient economic and political power to consider local economic development as a crucial policy goal. It does so by examining panel data including local party–political developments in the composition of the local government as well as variables measuring regional development to examine how neighboring SMSTs, cantons, and larger cities influence local development.

Hybrid panel models consisting of within- and between-estimates support the assumptions that a differentiated view of economic development helps to understand the potential of local politics to shape it. The findings show that party politics only has the potential to shape the residential economy, and that there is a weak, but statistically significant negative effect on local economic development for a government with a lower share of left parties. While the models do not explain much in terms of the development of LT-/HT industries who are supposed to develop idiosyncratic, KIBS are mainly driven by proximity to larger metropolitan centers.

Governments with a higher share of left parties more successfully provide an attractive environment for the residential sector, which is highly dependent on local demand. In contrast, the LT-/HT industries and KIBS are export oriented and interesting environments and attractive economic conditions interest them more. The results indicate that different party compositions in the local executive can lead to different local economic policy strategies that aim to support the residential economy. However, the paper does not measure concrete policy strategies and whether noneffects in KIBS stem from the considerable impact of regional developments so that local party–political differences in local economic strategies do not matter or from the fact that all parties agree on the same strategies should be subject to further research. Additionally, the paper captures local development only using the development of employment, although there are also other, equally important aspects that are relevant for local development, such as the number of firms, satisfaction of the population, or population growth. It would be interesting to see whether local autonomy and interdependences are similar when considering these aspects.

By analyzing a full sample of SMSTs, this paper offers a broad view of the relevance of local autonomy and party politics on local economic development. Since SMSTs are by definition municipalities of economic and political importance at least at regional levels, the findings can possibly also be generalized to smaller municipalities: when local party–politics does not affect the development of LT-/HT sectors and KIBS, it is likely that the effect holds true for smaller, more vulnerable municipalities with lower populations and fewer jobs.

The findings are also relevant beyond the Swiss context. This polycentric structure in a highly federalist setting provides the local level with very high autonomy and makes Switzerland a most likely case for local party–political influence on local economic development. If different political perspectives of how to shape the local economic structure only manifest themselves in the residential economic development, and weakly at that, it is likely that local party politics also has a low impact in other countries that have less local autonomy. On the one hand, the impact of regional and urban developments indicates that regional cooperation in different policy areas, such as economic development, land-use or transport policy, is crucial for local economic development in a federalist setting with high local autonomy and a competitive inter-municipal relationship (see also Sager 2005; Feiock, Steinacker, and Park 2009; Koch 2013; Wittwer 2020). On the other hand, the low impact of local party–political differences on the growth of employment indicates a post-political consensus on the need for economic growth, which classical critical urban theory criticizes (Harvey 1989).

A differentiated understanding of co-determination in economic development on different administrative levels can also contribute to regional institutionalism arguments and the “dilemma of scale” in democratic theory (Kantor 2006; Swanstrom 2006). This paper shows how local autonomy in economic development is limited by regional development, depends on the economic sector and therefore raises the question of how to deal with this regional influence from a local democratic perspective. Highlighting interdependencies can “represent opportunities for subnational authorities to develop new forms of democratic participation as well as new forms of organization” (Loughlin, Hendriks, and Lidström 2011, 10). If local politics seeks to have a democratically legitimized grip on local economic development in a federalist setting, it is worth noting that regional determinism and engagement in regional organizations can undermine local democratic entities. Matching democratic structures and economic functional regions would require consolidated regions that possess a regional institutionalized political entity or a regional activity democratically legitimized by local direct democratic procedures.

## Supplemental Material

sj-pdf-1-uar-10.1177_10780874211056519 - Supplemental material for How Strong is Local Politics’ Grip on Local Economic Development? The Case of Swiss Small and Medium-Sized Towns Click here for additional data file. Supplemental material, sj-pdf-1-uar-10.1177_10780874211056519 for How Strong is Local Politics’ Grip on Local Economic Development? The Case of Swiss Small and Medium-Sized Towns by Stefan Wittwer in Urban Affairs Review

## Notes

1 See operationalization table in the Appendix. The data points were selected because we only have employment data for these years.

2 As Meili and Mayer (2017) state, the measure of the economic sectors changed in 2008. However, since the correlation over time and for different measures in the same year remain very high (*Pearson’s r = *0.9998, *P *<* *.001), and we can control for year fixed effects, we also include data prior to 2008.

3 Solothurn is a strong outlier with 30 seats, while all the other SMSTs have less than 10 seats (with the exception of Sion and Grenchen with 15 seats).

4 See Figure A2 in the online Appendix for an illustration of the difference.

5 We used the newspaper online archives of swissdox and Factiva by searching for “[*name of SMST*] AND wahl*”, “[*name of SMST*] AND élect*” and “[*name of SMST*] AND elez*”.

6 Other local left parties include mainly local Green parties that are not part of the GPS. Those parties are the ones with a stronger focus on redistribution and social justice in Switzerland.

7 A total of 20 min is a good proxy for a commuting distance indicating closeness (Kloosterman and Musterd 2001). Within Swiss cities, reaching distances within cities also easily requires more than 20 min of driving time.

8 For the development prior to 2008, we rely on the cantonal aggregate income that has been measured until 2008. After 2008, the measurement of the FSO has been changed to the gross domestic product of cantons (see operationalization table).

9 For the cantons of Basel-City and Basel-Land, I calculated an average value for both cantons, since the canton of Basel-Land lies in the metropolitan area of the city of Basel and they cooperate intensely.

10 The lagged dependent variable ensures that the models consider whether prior development in a specific economic sector (growth or shrinkage) impacts development two time periods later, instrumenting *t − 1*. Excluding the lagged dependent variable does not substantially alter the results of the other variables (see Table A4 in the online Appendix). The effect of prior development therefore varies considerably between SMSTs and is on average, if at all, only weakly positive. This indicates that the dataset is not very dynamic and that including the lag does not lead to a bias in the results due to autocorrelation.

11 Table A6 in the appendix also reports marginal effects of spatial panel models for the four models with spatial lags (i.e., development in neighbouring SMSTs), which do not systematically differ from the hybrid models in Table 2.

12 If the lines that display the confidence intervals do not cross the vertical line of 0, they are statistically significant at the 90% level.
