# Supplementary material for: How Strong is Local Politics’ Grip on Local Economic Development? The Case of Swiss Small and Medium-Sized Towns
Source: Urban Aff Rev Thousand Oaks Calif. 2021 Nov 30;59(2):476–505. doi: 10.1177/10780874211056519 (PMC9893308; doi:10.1177/10780874211056519)
Supplement: sj-pdf-1-uar-10.1177_10780874211056519 - Supplemental material for How Strong is Local Politics’ Grip on Local Economic Development? The Case of Swiss Small and Medium-Sized Towns [file sj-pdf-1-uar-10.1177_10780874211056519.pdf]

Online Appendix

1. List of Swiss SMSTs (page 2)
2. Figure illustration the distribution of the number of neighbours (page 3)
3. Table with regressions with full employment (all sectors) as dependent variable (page 4)
4. Table with results for regressions with models without a lagged dependent variable and a brief discussion (page 5)
5. Tables with results of fixed-effects models estimating the effect of prior economic development on local political development and a brief discussion (page 6)
6. Table A6 that reports marginal effects using spatial panel regressions (page 7)
7. Figure illustrating the relationship of local vote share in national parliamentary elections and the composition of the local government (page 8).
8. Figure illustrating the effects of institutional context on the role of local party-politics in local economic development (page 9).

Table A2: List of Swiss SMSTs

Municipality	Canton
Affoltern am Albis	ZH
Bassersdorf	ZH
Bülach	ZH
Kloten	ZH
Opfikon	ZH
Wallisellen	ZH
Regensdorf	ZH
Hinwil	ZH
Rüti (ZH)	ZH
Wetzikon (ZH)	ZH
Adliswil	ZH
Horgen	ZH
Richterswil	ZH
Thalwil	ZH
Wädenswil	ZH
Küsnacht (ZH)	ZH
Männedorf	ZH
Meilen	ZH
Stäfa	ZH
Zollikon	ZH
Illnau-Effretikon	ZH
Pfäffikon	ZH
Dübendorf	ZH
Uster	ZH
Volketswil	ZH
Dietikon	ZH
Schlieren	ZH
Urdorf	ZH
Lyss	BE
Langenthal	BE
Köniz	BE
Muri bei Bern	BE
Zollikofen	BE
Ittigen	BE
Ostermundigen	BE
Burgdorf	BE
Münchenbuchsee	BE
Interlaken	BE
Münsingen	BE
Spiez	BE
Belp	BE
Steffisburg	BE
Thun	BE
Emmen	LU
Ebikon	LU
Horw	LU
Kriens	LU
Sursee	LU
Altdorf (UR)	UR
Einsiedeln	SZ
Freienbach	SZ
Arth	SZ
Schwyz	SZ
Sarnen	OW
Stans	NW
Baar	ZG
Cham	ZG
Risch	ZG
Steinhausen	ZG
Zug	ZG
Bulle	FR
Fribourg	FR
Villars-sur-Glâne	FR
Grenchen	SO
Olten	SO
Solothurn	SO
Riehen	BB
Aesch (BL)	BB
Allschwil	BB
Arlesheim	BB
Binningen	BB
Birsfelden	BB
Münchenstein	BB
Muttenz	BB
Oberwil (BL)	BB
Reinach (BL)	BB
Liestal	BB
Pratteln	BB
Neuhausen am Rheinfall	SH
Schaffhausen	SH
Herisau	AA
Rorschach	SG
Altstätten	SG
Buchs (SG)	SG
Rapperswil-Jona	SG
Flawil	SG
Uzwil	SG
Wil (SG)	SG
Gossau (SG)	SG
Davos	GR
Chur	GR
Aarau	AG
Suhr	AG
Baden	AG
Spreitenbach	AG
Wettingen	AG
Wohlen (AG)	AG
Brugg	AG
Lenzburg	AG
Möhlin	AG
Rheinfelden	AG
Oftringen	AG
Zofingen	AG
Arbon	TG
Romanshorn	TG
Amriswil	TG
Frauenfeld	TG
Kreuzlingen	TG
Weinfelden	TG
Bellinzona	TI
Locarno	TI
Chiasso	TI
Mendrisio	TI
Aigle	VD
Crissier	VD
Prilly	VD
Pully	VD
Renens (VD)	VD
Bussigny	VD
Ecublens (VD)	VD
Morges	VD
Gland	VD
Nyon	VD
Payerne	VD
Montreux	VD
La Tour-de-Peilz	VD
Vevey	VD
Yverdon-les-Bains	VD
Brig-Glis	VS
Martigny	VS
Monthey	VS
Sierre	VS
Sion	VS
Visp	VS
La Chaux-de-Fonds	NE
Le Locle	NE
Neuchâtel	NE
Carouge (GE)	GE
Chêne-Bougeries	GE
Le Grand-Saconnex	GE
Lancy	GE
Meyrin	GE
Onex	GE
Plan-les-Ouates	GE
Thônex	GE
Vernier	GE
Versoix	GE
Delémont	JU

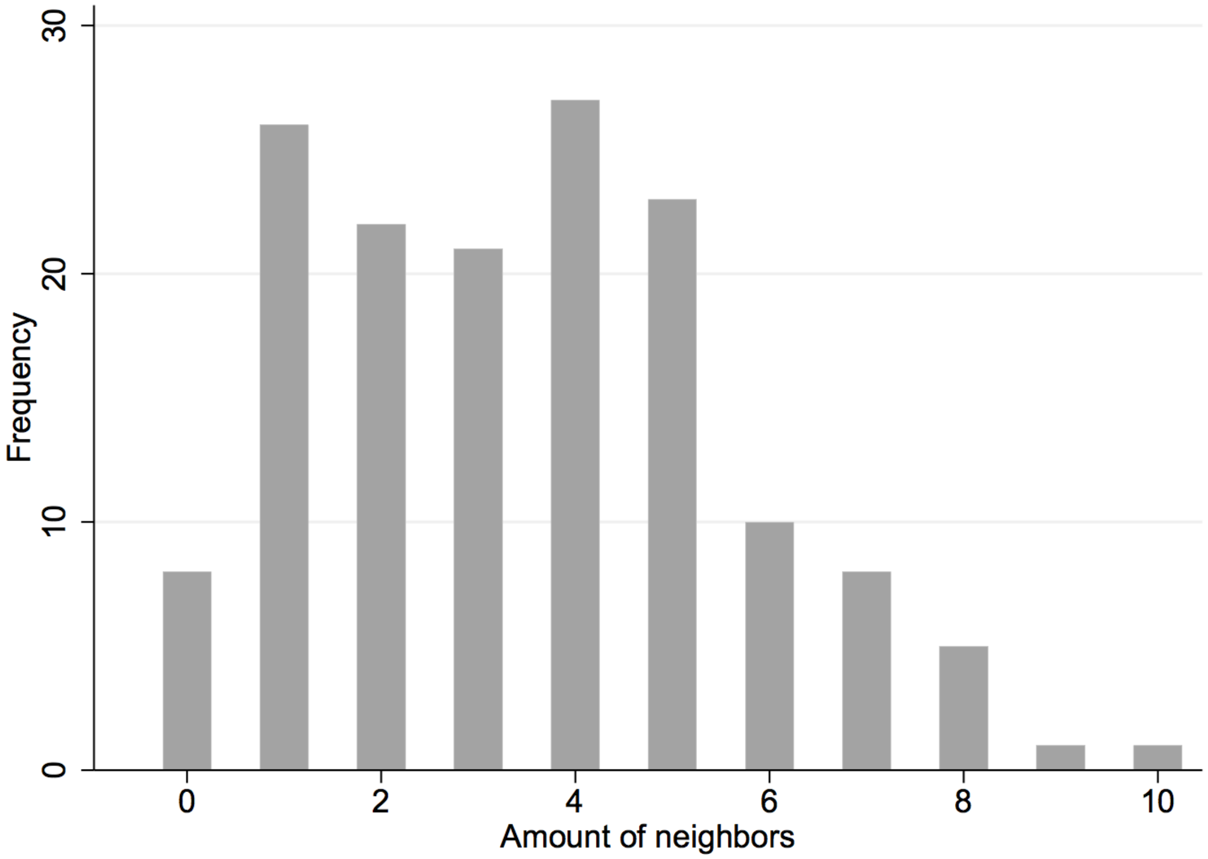

Figure A1: Distribution of number of neighbours

Table A3 for regressions with full employment (all sectors) as dependent variable.

	(1) Employment	(2) Employment	(3) Employment
Lagged DV	0.166*** (0.0412)	0.147*** (0.0392)	0.156*** (0.041)
Party composition (*between*)	-0.210 (0.336)	-0.206 (0.335)	-0.210 (0.335)
Party composition (*within*)	-0.0795* (0.0414)	-0.0731* (0.0398)	-0.0772* (0.0409)
Economic dev. Neighbors (*within*)		0.126*** (0.0406)	
Cantonal economic Development (*within*)			0.087* (0.046)
Distance to metro-politan centre (*between*)	-0.0606*** (0.0215)	-0.0603*** (0.0216)	-0.0606*** (0.0215)
Population (*between*)	0.852*** (0.0864)	0.853*** (0.0865)	0.852*** (0.0865)
Population (*within*)	0.317*** (0.0910)	0.308*** (0.0860)	0.327*** (0.0988)
Cantonal and year fixed effects	Yes	Yes	Yes
Intercept	8.542*** (0.308)	8.545*** (0.308)	8.576*** (0.308)
N	740	740	740
R^2 *within*	0.68	0.69	0.70
R^2 *between*	0.56	0.56	0.54

Cluster-robust standard errors in parentheses
* $p < 0.1$, ** $p < 0.05$, *** $p < 0.01$

Table A4: Results for models without lagged dependent variable

	(1.1)<br>Res	(2.1)<br>LT	(3.1)<br>HT	(4.1)<br>KIBS	(1.2)<br>Res	(2.2)<br>LT	(3.2)<br>HT	(4.2)<br>KIBS
**Lagged DV (*t-2*)**	**No**	**No**	**No**	**No**	**No**	**No**	**No**	**No**
Party composition (*between*)	-0.102<br>(0.341)	-0.159<br>(0.629)	-0.945<br>(1.145)	-0.567<br>(0.638)	-0.107<br>(0.342)	-0.160<br>(0.628)	-0.945<br>(1.147)	-0.582<br>(0.641)
Party composition (*within*)	-0.0882**<br>(0.0393)	-0.258<br>(0.187)	0.196<br>(0.280)	-0.0702<br>(0.199)	-0.0943**<br>(0.0399)	-0.246<br>(0.188)	0.179<br>(0.279)	-0.0928<br>(0.202)
Economic dev. Neighbors (*within*)	0.150***<br>(0.0458)	-0.0276<br>(0.115)	0.0477<br>(0.250)	0.457**<br>(0.208)				
Cantonal econ Development (*w*)					0.0959*<br>(0.0582)	0.256*<br>(0.135)	-0.372*<br>(0.225)	0.190<br>(0.227)
Distance to metro-politan centre (*between*)	-0.070***<br>(0.0236)	0.00303<br>(0.0360)	0.00992<br>(0.0828)	-0.213***<br>(0.0390)	-0.070***<br>(0.0235)	0.00311<br>(0.0360)	0.00977<br>(0.0827)	-0.214***<br>(0.0389)
Population (*between*)	0.971***<br>(0.0904)	0.697***<br>(0.149)	0.631**<br>(0.308)	1.078***<br>(0.173)	0.971***<br>(0.0904)	0.697***<br>(0.149)	0.630**<br>(0.308)	1.077***<br>(0.173)
Population (*within*)	0.431***<br>(0.0824)	0.242<br>(0.219)	0.915**<br>(0.459)	0.550*<br>(0.282)	0.458***<br>(0.0949)	0.256<br>(0.223)	0.895**<br>(0.429)	0.626**<br>(0.309)
Year and cantonal fixed effects	Yes	Yes	Yes	Yes	Yes	Yes	Yes	Yes
N	740	740	740	740	740	740	740	740
R^2 *within*	0.76	0.04	0.02	0.23	0.76	0.04	0.02	0.22
R^2 *between*	0.58	0.34	0.26	0.41	0.58	0.34	0.26	0.41

Cluster-robust standard errors in parentheses
* $p < 0.1$, ** $p < 0.05$, *** $p < 0.01$

Note to table A4: The results of the models without the lagged dependent variable measuring previous development in the respective sectors show, that the inclusion of the lagged dependent variable does not change the models significantly (not regarding significance and number of the coefficients and also not regarding R^2).

Table A5: Fixed-effects models estimating the effect of prior economic development on local political development

	(1) Political composition (Effect of Res)	(2)	(3) Political composition (Effect of MHT)	(4)	(5) Political composition (Effect of KIBS)	(6)	(7) Political composition (Effect of MLT)	(8)
Lagged DV (t-1)		0.353*** (0.0631)		0.362*** (0.0626)		0.360*** (0.0633)		0.358*** (0.0639)
Prior development of Res	-0.0814 (0.0587)	-0.0933 (0.0584)						
Prior development of MLT			-0.00404 (0.0255)	-0.00079 (0.0313)				
Prior development of MHT					0.0170 (0.0109)	0.0114 (0.0102)		
Prior development of KIBS							-0.0206 (0.0125)	-0.0198 (0.0135)
Population	-0.00042 (0.00091)	0.00036 (0.0009)	-0.0008 (0.00085)	0.00001 (0.0008)	-0.00092 (0.00085)	-0.00009 (0.0008)	-0.00072 (0.00084)	0.000056 (0.0008)
Year fixed effects	Yes	Yes	Yes	Yes	Yes	Yes	Yes	Yes
N	888	740	888	740	888	740	888	740

Standard errors in parentheses
* $p < 0.1$, ** $p < 0.05$, *** $p < 0.01$

Note: Table A5 addresses potential endogeneity in the variable measuring political change. Does political change depend on prior economic development? The models in table 7 are in line with recent research arguing that political preferences are not affected by volatile economic circumstances (O’Grady 2019) or local economic conditions and tax rates (James and John 2006; Elinder 2010)[1] and hence show that the measure of the local party composition is not endogenous to economic development. The respective first models include the available years of 2001 to 2013 while the respective second models include a lagged dependent variable controlling for prior political change at the local level. The results do not alter the variables of interest.

[1] See main document for references.

Table A6: Reported output for marginal effects using spatial panel regressions

	(1)<br>Res	(2)<br>LT	(3)<br>HT	(4)<br>KIBS
Prior development<br>in the sector (*t-2*)	0.135*** (0.0277)	0.121*** (0.0296)	0.0495<br>(0.0318)	0.0927*** (0.0335)
Party composition<br>(*between*)	-0.101<br>(0.289)	-0.157<br>(0.517)	-0.942<br>(0.997)	-0.568<br>(0.570)
Party composition<br>(*within*)	-0.0798*** (0.0299)	-0.290*** (0.0903)	0.184<br>(0.176)	-0.0741<br>(0.150)
Economic development of<br>Neighbours (*within*)	0.114*** (0.0394)	-0.0227<br>(0.117)	0.0615<br>(0.229)	0.403** (0.196)
Distance to metropolitan<br>centre<br>(*between*)	-0.0698*** (0.0266)	0.00308<br>(0.0476)	0.00989<br>(0.0919)	-0.213*** (0.0525)
Population<br>(*between*)	0.971*** (0.0947)	0.697*** (0.169)	0.630* (0.326)	1.078*** (0.187)
Population<br>(*within*)	0.351*** (0.0662)	0.237<br>(0.193)	0.859** (0.380)	0.536* (0.322)
N	740	740	740	740

Average marginal effects (dy/dx) reported, standard errors in parentheses
* $p < 0.1$, ** $p < 0.05$, *** $p < 0.01$

Note: The results of the models using spatial panel regressions are very similar to the hybrid panel models. However, with the hybrid panel models, it is possible to include cluster-robust standard errors. The different standard errors for the party composition in model 2 and on prior development in model 4 would also be similar to the spatial panel regressions when not using cluster-robust standard errors in the hybrid panel models.

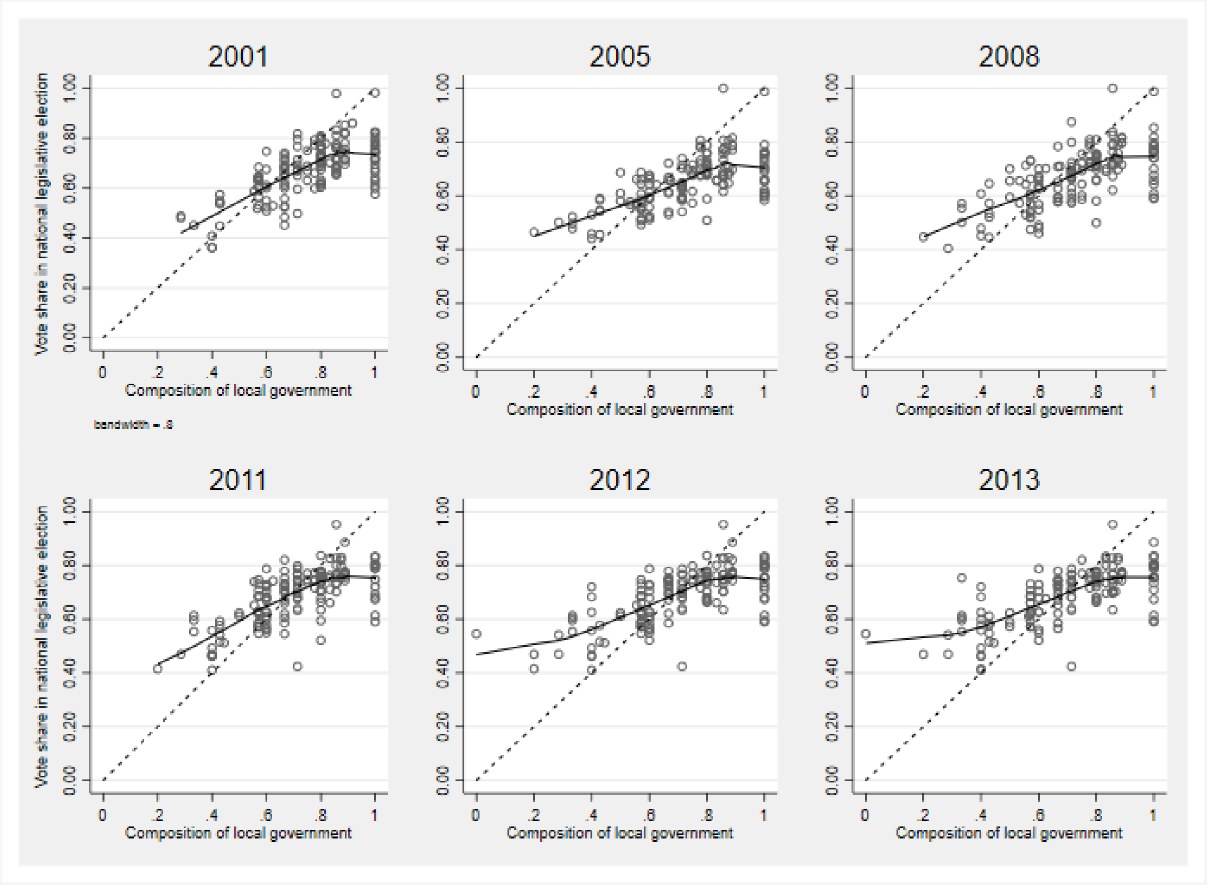

Figure A2: Relationship of local vote share in national parliamentary elections and the composition of the local government. The solid line indicates locally weighted scatterplot smoothing (LOWESS), the dashed line indicates same values for both measures.

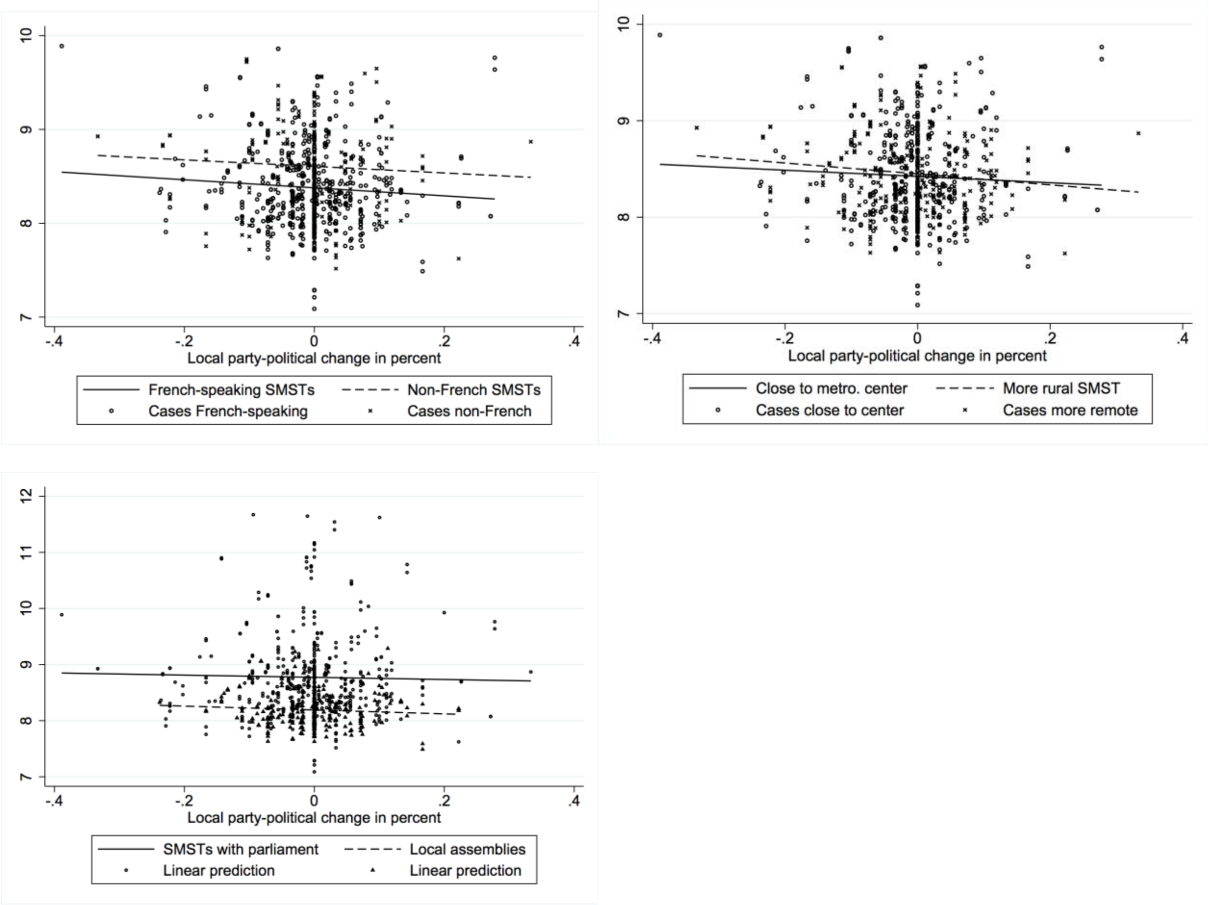

Figures A3: The figures show the effects of local party-political change on the development of the residential economy (the predicted effects from Model 1) – the economic sector where we see and overall effect. The comparison of the coefficients for local party-political change for SMSTs with a parliament and those without, as well as the coefficients for change for SMSTs in French-speaking SMSTs and the others and the coefficients for SMSTs close to a metropolitan centre and those more remote shows that the coefficients have a similar slope. If we include the dummies in the Models with an interaction effect on local party-political change, the interactions all are neither significant nor substantial.
